# Effects of pneumoperitoneum on kidney injury biomarkers: A randomized clinical trial

**DOI:** 10.1371/journal.pone.0247088

**Published:** 2021-02-19

**Authors:** Marcos Antonio Marton Filho, Rodrigo Leal Alves, Paulo do Nascimento, Gabriel dos Santos Tarquinio, Paulo Ferreira Mega, Norma Sueli Pinheiro Módolo

**Affiliations:** 1 Department of Surgical Specialties and Anesthesiology, Botucatu School of Medicine, Universidade Estadual Paulista (UNESP), Botucatu, São Paulo, Brazil; 2 Centro Universitário Estácio de Ribeirão Preto, Ribeirão Preto, São Paulo, Brazil; 3 Department of Anesthesiology, São Rafael Hospital and Federal University of Bahia, Salvador, Brazil; 4 Clínica de Anestesiologia de Ribeirão Preto (CARP), Ribeirão Preto, São Paulo, Brazil; 5 School of Medicine, Pontifícia Universidade Católica de Campinas (PUC-CAMPINAS), Campinas, São Paulo, Brazil; Cleveland Clinic, UNITED STATES

## Abstract

**Background:**

Increased intra-abdominal pressure causes hemodynamic changes that may affect renal biomarkers.

**Methods:**

This randomized, single-blind, single-center clinical trial recruited patients undergoing laparoscopic cholecystectomy at a tertiary care center in Brazil. They were randomly allocated to a standard intra-abdominal pressure group (P_10-12_, 10–12 mm Hg) and a low intra-abdominal pressure group (P_6-8_, 6–8 mm Hg). The primary outcome was the change in neutrophil gelatinase-associated lipocalin (NGAL) and cystatin C levels measured at the beginning of the procedure (T0), at the end of the procedure (T1), and 24 hours after the procedure (T2). P-values < 0.05 were considered statistically significant.

**Results:**

In total, 64 patients completed the study—33 were given standard pressure and 31 were given low pressure. There was no significant difference in the biomarker between the groups (*P* = 0.580), but there was a significant difference between the time points with elevation at T1 (*P* < 0.001). Similar to NGAL, cystatin C had an elevation at T1 in both groups (*P* = 0.021), but no difference was found when comparing the groups.

**Conclusions:**

In laparoscopic cholecystectomy, pneumoperitoneum increases NGAL and cystatin C levels intraoperatively, and the use of low-pressure pneumoperitoneum does not change the course of these biomarkers.

## Introduction

Laparoscopic surgery, introduced in 1987 with a successful cholecystectomy performed by Phillipe Mouret, and, more recently, robotic surgery have promoted a major revolution in many surgical specialties. Considered to be less invasive than traditional techniques, laparoscopy has lower rates of pain and complications and allows an early return to normal physiological functions. However, such procedures also pose new challenges, including the insufflation of an inert gas into the peritoneal cavity to create an adequate surgical field.^1^ The immediate consequences of insufflation are physiological changes in the cardiovascular and pulmonary systems and in the perfusion of abdominal viscera (especially kidney and liver). To minimize these changes, there are several guidelines on safe intra-abdominal pressure levels, which as a rule should not exceed 12 to 15 mm Hg, as well as on the management of anesthetic drugs and intraoperative blood volume [1].

Cystatin C is a low-molecular-weight protein (13.36 KDa) produced by nucleated cells at a constant rate whose function is to inhibit cysteine proteinases. It is freely filtered by the glomerulus, reabsorbed and metabolized in the proximal renal tubule, but has no renal or extrarenal secretion. It reflects, therefore, glomerular filtration only. Neutrophil gelatinase-associated lipocalin (NGAL) is a 25 KDa preapoptotic molecule originally characterized in neutrophils bound to gelatinase and mainly expressed in epithelial cells, thus in renal tubules. In addition to showing early elevation, it has a good performance in patients with severe renal injury and acute tubular necrosis, reaching a peak within approximately 3 hours and remaining elevated in this population for approximately 24 hours. In patients who do not progress with acute kidney injury, NGAL decreases after 1 hour of injury [2]. Thus, it has been shown to be a good predictor of indication for hemodialysis [3].

To assess the effects of pneumoperitoneum at different pressure levels on renal physiology of patients undergoing laparoscopic cholecystectomy, this randomized clinical trial compared NGAL and cystatin C values obtained before and after intervention under pressures of 6 to 8 mm Hg in the low pressure group (P_6-8_) and 10 to 12 mm Hg in the standard pressure group (P_10-12_).

## Methods

This randomized, prospective, controlled clinical trial was conducted over 2 consecutive years at a tertiary care hospital in southeastern Brazil. The study was approved by the Research Ethics Committee at the Botucatu Medical School with protocol no. 2.230.721, Certificate of Presentation for Ethical Consideration (CAAE) no. 71370517.1.0000.5411 on August 21, 2017, and was registered at the Brazilian Registry of Clinical Trials (REBEC) with identification no. RBR-5tyrtt in December 2017. The study was reported according to the Consolidated Standards of Reporting Trials (CONSORT) [4]. All participants signed an informed consent form. Patients were recruited from January 2018 to January 2020.

Patients aged 18 years or over, of both sexes, with clinical indication for cholecystectomy were eligible for the study. Exclusion criteria were estimated creatinine clearance < 60 mL/min (using the Cockcroft-Gault equation) in preanesthetic assessment, body mass index (BMI) > 35 kg/m^2^, formal indication for open surgery, anesthetic contraindication due to drug allergy, or inadequate clinical conditions for the anesthesia protocol.

### Randomization and blinding

To maintain the number of participants proposed for each group, randomization was conducted in blocks of six individuals using a randomization software. This was performed in order to minimize imbalances of potential confounding variables. For each block, three sealed envelopes containing information regarding allocation to either P_6-8_ or to P_10-12_ group were generated and distributed to the medical researchers directly involved in the medical procedure. Those envelopes were to be open at the operation theatre. The surgeon was not blinded. The patient was not informed of the allocated group. The researches involved in the data analyses and randomization process did not participated in the medical care.

### Anesthesia protocol and surgery

Both groups underwent total intravenous anesthesia with midazolam, remifentanil, and target-controlled propofol. Hydration was performed with Ringer’s lactate using 10 mL/kg ideal body weight in the first hour, 8 mL/kg ideal body weight in the second and third hours, and 6 mL/kg ideal body weight in the subsequent hours. Dexamethasone was used as an antiemetic, and cefazolin was used as antibiotic prophylaxis. Patients were monitored for vital signs, diuresis, blood glucose, and ventilatory parameters. Neuromuscular blockade was obtained with administration of atracurium and monitored with a train of four (TOF) targeted at 0 and a posttetanic count (PTC) also targeted at 0. Postoperative analgesia was performed with dipyrone or acetaminophen and rescue morphine. Hemodynamic and ventilatory parameters were monitored throughout the anesthetic procedure.

Both groups had a pneumoperitoneum created, and intra-abdominal pressure was then maintained with values ranging from 6–8 mm Hg in the P_6-8_ group and 10–12 mm Hg in the P_10-12_ group. Then, four abdominal points were punctured, and the gallbladder was removed according to each patient’s conditions. At the end of the procedure, the entire pneumoperitoneum was undone.

Blood samples were collected immediately after the first venoclysis (time point 0, T0), at the end of the anesthetic procedure (T1), and 24 hours after the end of the procedure (T2).

### Laboratory analysis

The samples were promptly centrifuged, and the plasma was stored in a freezer at 80°C for conservation. NGAL was measured using the Human NGAL (Neutrophil Gelatinase Associated Lipocalin) ELISA Kit (Catalog No: E-EL-H0096) and cystatin C was measured using Human Cys-C (Cystatin C) Elisa Kit (Catalog No: E-EL-H3643), both from Elabscience^®^. Dosages were made in the institution’s laboratory.

### Statistical analysis

Continuous demographic variables were assessed using the Shapiro-Wilk test for normality. Those with normal distribution were reported as mean and standard deviation, while those with non-normal distribution were reported as median and quartiles. Categorical variables were described as absolute and relative frequencies. For quantitative variables that had normal distribution, we used the Student’s t-test, while for those that did not, we used the Mann-Whitney U test. To compare NGAL and cystatin C values at the 3 different time points, we used repeated-measures analysis of variance (ANOVA) with Bonferroni correction after confirmation of normal distribution (Shapiro-Wilk test) and homogeneity of variance (Levene’s test). For the main outcomes, the differences between groups and the 95% confidence intervals (CI) were also calculated for differences between means (t test and ANOVA) and medians (Hodges Lehmann estimative), as appropriated. The significance level was set at 5%.

### Sample size calculation

For sample size calculation, we used as statistical parameters an expected difference in NGAL values between the groups of about 6.0 ng/dL and standard deviation of 8.4 ng/dL based on the literature [5], with a power of 80% and a significance level of 5%. Thus, 31 patients were required per group.

## Results

**Fig 1:**
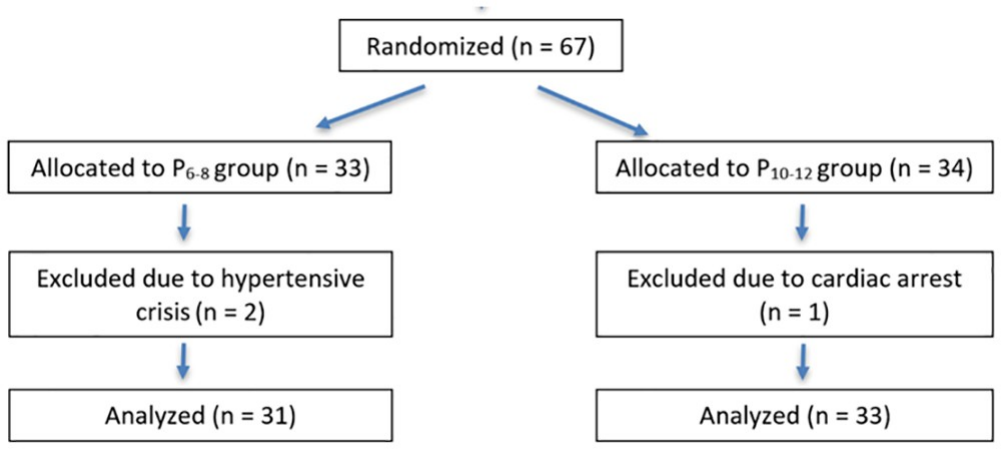
Study flowchart. Population selection, randomization and analysis.

We initially selected 74 patients, and 64 completed the study (Fig 1), of which 50 were female and 14 were male (Table 1). We found no differences in perioperative monitoring data between the groups. No patient presented hemodynamic instability. Intraoperative blood glucose, although statistically different, remained within the normal intraoperative range.

**Table 1:** Demographic and anthropometric variables.

	P_6-8_ group (n = 33)	P_10-12_ group (n = 34)
Age (years)^†^	49.6 ± 13.2	44.4 ± 13.5
Male^††^	22.6%	21.2%
Weight (kg)^†^	73.8 ± 10.5	74.7 ± 11.8
Height (cm)^†^	162.4 ± 9.9	164.3 ± 7.5
Body mass index (kg.m^-2^)^†^	27.9 ± 3.3	27.6 ± 4.2

^†^Values reported as mean and standard deviation in each study group.

^††^Values reported as relative frequency (chi-square test).

Two participants were excluded from the study because they had a hypertensive crisis, requiring the use of drugs not provided for in the protocol. One participant had a cardiac arrest in the post-anesthesia recovery room due to respiratory depression. As we expected, mechanical ventilation parameters were significantly different between the groups (Table 2) given the variation in pneumoperitoneum pressure (higher peak pressure and positive end-expiratory pressure [PEEP] values in the P_10-12_ group).

**Table 2:** Perioperative variables.

	P_6-8_ group (n = 33)	P_10-12_ group (n = 34)	*P* value
Pneumoperitoneum time (minutes)^†^	77 ± 33	70 ± 15	0.382
Duration of anesthesia (minutes)^‡^	131 ± 26	118 ± 27	0.139
Arterial blood oxygen saturation* (%)^‡^	100 (100–100)	100 (99–100)	0.333
Mean blood pressure* (mm Hg)^‡^	80 (72–93)	83 (79–92)	0.295
ETCO_2_* (mm Hg)^‡^	35 (32–37)	35 (33–38)	0.336
Blood glucose* (mg/dL)^‡^	110 (95–132)	97 (87–104)	0.011
Peak pressure* (cmH_2_O)^‡^	19 (17–21)	22 (19–23)	0.006
Plateau pressure* (cmH_2_O)^‡^	17 (15–19)	20 (18–24)	0.001
PEEP* (cmH_2_O)^‡^	5 (5–5)	5 (5–5)	0.121

*Values referring to 1 hour after the beginning of the procedure.

^†^Values reported as mean and standard deviation in each study group.

^‡^Values reported as median and first and third quartiles (Mann-Whitney U test and Hodges Lehmann estimative).

ETCO_2_, end-tidal carbon dioxide; PEEP, positive end-expiratory pressure.

When we compared time points, NGAL showed a similar behavior in both groups—significantly increased at T1, returning to baseline values at T2 (24 hours after the procedure), i.e., there was no significant difference between T0 and T2. For cystatin C at the different time points, we found a small increase in T1 and a subsequent reduction, with a significant difference between T1 and T2 (Table 3).

**Table 3:** Comparison of NGAL and cystatin C values at different perioperative time points.

Marker	T0	T1	T2	Differences between means with 95% CI
NGAL* (ng/dL)	60.4 ± 11.3	153.6 ± 19.3	62.3 ± 10.4	T0—T1: -93.1 (-98.3 to 87.9)
				T1—T2: 91.1 (85.4 to 96.9)
				T0—T2: -1.9 (-1.1 to -5.0)
Cystatin C* (ng/dL)	2324.3 ± 540.8	2677.7 ± 927.4	2384.2 ± 500.6	T0—T1–353.3 (-620.9 to -85.6)
				T1—T2 293.4 (-94.9 to 681.9)
				T0—T2–59.8 (-289.9 to 170.2)

NGAL, neutrophil gelatinase-associated lipocalin.

95% CI, 95% confidence interval for the difference between means.

*Values reported as mean and standard deviation.

P value for NGAL comparisons <0.001 (repeated measure ANOVA).

P value for Cystatin C comparisons 0.021 (repeated measure ANOVA).

T0, beginning of the procedure; T1, at the end of the anesthetic procedure; T2, 24 hours after the end of the procedure.

When comparing the groups, in turn, we found no significant difference in any of the study time points for either NGAL (Table 4) or Cystatin C (Table 5).

**Table 4:** Comparison of the effect of the groups on neutrophil gelatinase-associated lipocalin (NGAL, ng/dL) values at different time points.

Group	T0	T1	T2	Differences between groups at each time with 95% CI
**P**_**6-8**_ (n = 33)*	60.8 ± 11.1	148.3 ± 17.8	63.9 ± 8.8	T0: -0.7 (-6.9 to 5.5)^a^
**P**_**10-12**_ (n = 34)*	60.1 ± 11.5	157.5 ± 19.8	61.2 ± 11.4	T1: 9.2 (-1.4 to 19.8)^b^
**Estimated mean**^†^	60.5 ± 1.6	152.9 ± 2.7	62.5 ± 1.4	T2: -2.7(-8.4 to 3.0)^c^

P_6-8_ –Low pneumoperitoneum pressure; P_10-12_ –Standard pneumoperitoneum pressure.

95% CI– 95% Confidence interval for the difference between means.

*Values reported as mean and standard deviation.

^†^Values reported as estimated mean and standard error.

^a^ difference between means of NGAL in P_6-8_ and P_10-12_ at T0.

^b^ difference between means of NGAL in P_6-8_ and P_10-12_ at T1.

^c^ difference between means of NGAL in P_6-8_ and P_10-12_ at T2.

P value for differences between groups 0.580 (Repeated measure ANOVA).

T0, beginning of the procedure; T1, at the end of the anesthetic procedure; T2, 24 hours after the end of the procedure.

**Table 5:** Comparison of the effect of the groups on cystatin C values (ng/dL) at different time points.

Group	T0	T1	T2	Differences between groups at each time with CI 95
**P**_**6-8**_ (n = 33)*	2184.9 ± 536.0	2631.6 ± 1092.6	2233.9 ± 527.2	T0: 244.0 (-55.8 to 543.8)^a^
**P**_**10-12**_ (n = 34)*	2428.9 ± 529.8	2712.2 ± 801.2	2496.9 ± 457.0	T1: 234.6 (-274.6 to 743.8)^b^
**Estimated mean**^†^	2306.9 ± 76.8	2671.9 ± 135.1	2365.4 ± 70.4	T2: 263.0 (-14.1 to 540.1)^c^

P_6-8_ –Low pneumoperitoneum pressure; P_10-12_ –Standard pneumoperitoneum pressure.

95% CI– 95% confidence interval for the difference between means.

*Values reported as mean and standard deviation.

^†^Values reported as estimated mean and standard error.

^a^ difference between means of cystatin C in P_6-8_ and P_10-12_ at T0.

^b^ difference between means of cystatin C in P_6-8_ and P_10-12_ at T1.

^c^ difference between means of cystatin C in P_6-8_ and P_10-12_ at T2.

P value for differences between groups 0.154 (Repeated measure ANOVA).

T0, beginning of the procedure; T1, at the end of the anesthetic procedure; T2, 24 hours after the end of the procedure.

## Discussion

Increased intra-abdominal pressure leads to a series of pathophysiological changes in arterial blood pressure, venous blood pressure, and visceral perfusion pressure with negative effects on most organs. Implementation of deep neuromuscular blockades [6, 7] and improvement in surgical material with enhanced video resolution and better control of injection, sealing, and pressure of the abdominal gas have allowed in recent years a gradual reduction in the pneumoperitoneum pressure required to perform a range of laparoscopic procedures.

Renal circulation is greatly affected by increased intra-abdominal pressure, and measurement of kidney injury biomarkers shows significant changes during pneumoperitoneum with a positive correlation with pressure levels [8]. A change in renal blood flow was suggested in a meta-analysis conducted by Wever et al, although the authors highlight the low quality of the evidence, the need for further studies to clarify the issue, and the fact that only animal studies were included [9]. Pneumoperitoneum was also associated with acute kidney injury in a study conducted by Srisawat et al, showing that insufflation duration and blood loss are increasingly important factors as procedures become more complex [10]. Thus, we chose to focus on the effect of pneumoperitoneum on renal function in patients undergoing cholecystectomy, a procedure that is simple to perform, produces no significant blood loss, and has short pneumoperitoneum time.

Hemodynamic control in the perioperative period and vigorous hydration are considered protective factors for the kidney, as Brienza et al demonstrated in a meta-analysis of studies in humans [11]. Our study included hydration with lactated Ringer’s solution following a strict protocol and continuous monitoring of hemodynamic parameters. Apart from renal changes, some authors have demonstrated the absence of a corresponding clinical effect for changes in biomarkers, as these were shown to be transient after pneumoperitoneum [12]. Although there is a lack of long-term follow-up studies, experimental evidence showed no permanent changes in renal function [9]. Although serum creatinine levels change slowly, this biomarker remains the most commonly used in clinical practice, as it is included in diagnostic scores such as RIFLE (Risk, Injury, Failure, Loss, End Stage Renal Disease) [13], AKIN (Acute Kidney Injury Network) [14], and, more recently, KDIGO (Kidney Disease: Improving Global Outcomes) [15]. To overcome a diagnostic delay due to a late increase in creatinine, there was an intense search for new biomarkers whose sensitivity and early change profile would allow an earlier diagnosis of the injury, among which we highlight NGAL and cystatin C.

Elevations in NGAL and cystatin C are caused by different mechanisms of injury to the nephron—elevated NGAL stems from tubular injury, while elevated cystatin C stems from impaired glomerular filtration, both considered possible changes in the event of decreased renal flow. Although they have a well-established role in early diagnosis of kidney injury, these new biomarkers and their behavior still need to be investigated in different clinical settings. There is evidence of their prognostic value in abdominal trauma [16], in the postoperative period of cardiac surgery [17], and in diseases such as chronic obstructive pulmonary disease [18]. Some authors have demonstrated that their levels increase up to 48 hours before creatinine [19], with appropriate performance to predict the need for renal replacement therapy, while others have reported no prognostic value [20]. In a recent cohort of patients undergoing cardiac surgery, cystatin C was superior to creatinine in predicting long-term mortality [21]. Cystatin C and NGAL also showed high sensitivity and specificity for renal function in kidney transplant recipients, having an important association with the prognosis of these patients [22, 23].

NGAL can be measured in both plasma and urine. Kiseli et al demonstrated slight changes in the urine marker, with no evident clinical significance [12]. We opted for the plasma marker because it suggested superiority in the prediction of acute kidney injury as reported by some authors [24, 25]. In our study, serum NGAL was shown to be an effective biomarker for acute kidney injury, with elevations observed at the end of the anesthetic procedure. The mean duration of this procedure was greater than 120 minutes, during which changes are usually detected. We also found a reduction in biomarker levels after 24 hours, as described in the literature. There is evidence showing that early return to baseline NGAL levels is predictive of favorable outcomes, while persistently high levels correlate with higher mortality [25].

Similar to NGAL, cystatin C also showed an elevation at the end of the surgery, which is consistent with the findings of a study conducted by Lima et al in patients undergoing laparoscopic cholecystectomy [26]. Thus, for a glomerular filtration marker, it is worth mentioning the protective role that adequate volume management may have during the procedure, as there is no impairment to the filtration rates measured by this method, despite any tubular endothelial injury shown by NGAL.

We attribute the lack of difference between the study groups to the short duration of the procedure (120 minutes) and to the pressure ranges (6–8 mm Hg and 10–12 mm Hg) used in the present study. Although our aim was to focus on pneumoperitoneum as the only factor in the study, such limitations may explain the lack of difference for different pressure ranges. In a study using NGAL in patients undergoing bariatric procedures with mean pressure of 12 mm Hg and pneumoperitoneum lasting approximately 130 minutes, Fernandes et al found no significant changes in the biomarker [27], which suggests that short duration is a relevant factor.

We also observed that although changes in renal blood flow due to pneumoperitoneum have been described in the literature, the kidney has transient changes with no corresponding clinical effect, as measured by sensitive markers for acute kidney injury, regardless of pressure levels as long as they respect the described limit of 12 mm Hg [7, 28].

## Conclusion

Patients undergoing laparoscopic cholecystectomy show increased NGAL and cystatin C levels immediately after surgery, returning to normal within 24 hours. The use of low-pressure pneumoperitoneum does not influence the course of these kidney injury biomarkers, as long as the top intra-abdominal values are 12 mm Hg.

## Supporting information

S1 Checklist (DOC)
